# Indicators for adequate diabetes care for the indigenous communities of Ecuador

**DOI:** 10.1111/hex.13643

**Published:** 2022-10-31

**Authors:** Jimmy Martin‐Delgado, Carla Tovar, Israel Pazmiño, Amelia Briones, Irene Carrillo, Mercedes Guilabert, José Joaquín Mira

**Affiliations:** ^1^ Instituto de Investigación e Innovación en Salud Integral, Facultad de Ciencias Médicas Universidad Católica de Santiago de Guayaquil Guayaquil Ecuador; ^2^ Hospital Luis Vernaza Junta de Beneficencia de Guayaquil Guayaquil Ecuador; ^3^ 14D06 District of Health Ministry of Health Macas Ecuador; ^4^ Health Zone Coordination #3 Ministry of Health Riobamba Ecuador; ^5^ Facultad de Medicina Pontificia Universidad Católica del Ecuador Quito Ecuador; ^6^ Coordinación de Calidad Secretaria Metropolitana de Salud Municipio de Quito Ecuador; ^7^ Health Psychology Department Miguel Hernández University Elche Spain; ^8^ Health District Alicante‐Sant Joan Alicante Spain; ^9^ Atenea Research Group Foundation for the Promotion of Health and Biomedical Research Sant Joan d'Alacant Spain

**Keywords:** diabetes, Ecuador, indicators, indigenous population, Latin America, quality criteria

## Abstract

**Introduction:**

Diabetes is the second leading cause of death in Ecuador, as 79% of the indigenous population live in rural areas that are difficult to access and have below‐average health resources. The objective of this study was to define person‐centred indicators to monitor the care received by patients with diabetes in the indigenous population.

**Method:**

Qualitative research combining three focus groups (with the participation of 10 patients and 18 professionals) to capture relevant information and Delphi to reach a consensus on the pertinence, relevance, and feasibility of a set of indicators was conducted. Two rounds of the Delphi technique were performed, with the participation of 64 professionals in the first round (90% response rate) and 34 in the second round (53% response rate).

**Results:**

A total of 23 indicators were identified which were distributed in the previously identified six dimensions (cosmovision, accessibility, adaptability to cosmovision, resources, equipment, community care, quality culture and results).

**Conclusions:**

The consensus on the set of indicators among all the participants in this study strengthened the results obtained. These indicators have considered the feasibility and relevance and aimed to achieve comprehensive person‐centred care for diabetes among the indigenous population in Ecuador and possibly the Andean community.

**Patient or Public Contribution:**

These indicators’ development included patients and caregivers since its conception. During the qualitative phase of this research, relevant information on cultural and social beliefs was gathered directly from the study population to achieve patient‐centred indicators for adequate diabetes care.

## 1 INTRODUCTION

Non‐communicable diseases are a challenge for all health systems and are a global health problem. They are the leading causes of illness and preventable premature deaths in the Americas and Ecuador. These pathologies undermine individual and family well‐being and threaten socioeconomic development because of the burden associated with them due to the marked increase in treatment costs.^1^ Mitigating, preventing, and reducing mortality from cardiovascular diseases and diabetes are part of the 2030 Sustainable Development Goals.^2^

The global prevalence of diabetes in adults (20–79 years) is estimated to be 9.3% (463 million people) in 2019, which is estimated to increase to 10.2% (578 million) by 2030.^3^ In addition, the prevalence in South and Central American countries is expected to increase by 27% between 2019 and 2030.^3^, ^4^ The economic cost of diabetes is estimated to be USD 65,216 million per year in Latin America and the Caribbean.^5^

In Ecuador, the prevalence of diabetes is 7.3%,^6^, ^7^ and it is the second leading cause of death, only after ischaemic heart disease, for which diabetes is a well‐known risk factor.^8^ Clinical practice guidelines specific to diabetes^9^ include clinical and analytical parameters for the control of the disease. Although this guideline is applied throughout Ecuador, it does not include indicators to monitor the care provided. In Ecuador, 7% of the population is indigenous, and 79% live in rural areas that are difficult to access.^10^ Due to the socioeconomic and cultural differences in this group of indigenous people, the prevalence of diabetes is disproportionately higher. According to the latest National Health and Nutrition Survey of Ecuador, the indigenous community is the least active among Ecuador's five predominant ethnic groups. Less than 3% perform some physical activity for at least 60 min a day, and 57% are overweight or obese.^11^ Likewise, 73% of this minority population does not have access to social security, and their health resources are below the national average.^12^

The indigenous population typically holds beliefs or has customs and lifestyles that are markedly different. In Ecuador, indigenous cosmovision interprets life in plenitude or well‐being as a harmonious realization between man and nature.^13^ This belief differs from the scientific perspective on the causes and approaches to diabetes and other chronic pathologies. Moreover, low‐income sources of this population negatively impact diet and accessibility to health facilities. Therefore, it is necessary to establish a dashboard to measure the quality of care received by this population. Thus, this study aimed to define indicators from the perspective of person‐centred care to monitor the care received by patients with diabetes in the indigenous people.

## 2 METHODS

This qualitative study conducted through focus groups comprising managers, professionals, and patients initially identified the quality criteria relevant to the indigenous community. The consensus on the quality criteria was reached using the Delphi technique. Finally, a consensus conference technique was employed to identify indicators to monitor these criteria. This study was approved by the Ethics Committee of the Hospital Clínica Kennedy of Guayaquil, Ecuador (HCK‐CEISH‐19‐0041).

### 2.1 Study population

The indigenous nationalities and people of Ecuador are collectivities that assume an ethnic identity based on their culture, institutions, and history that define them as the indigenous peoples and descendants of pre‐Hispanic societies. The Republic of Ecuador recognizes indigenous peoples and nationalities by defining itself as intercultural and plurinational in its political constitution. According to the last national census conducted in 2010, 1,018,176 people identified themselves as indigenous.

The Ecuadorian territory comprises 24 provinces and nine administrative zones. These zones must coordinate technical and organizational activities for the optimal functioning of state ministries. This study included participants from rural areas of Zone 3 (Central Andes and Pastaza).

### 2.2 Inclusion criteria

The inclusion criteria for the patients were those who used the Ministry of Public Health for at least a year; belonged to any sex, were over 18 years of age, were diagnosed with type 2 diabetes, without a history of ischaemic heart disease or stroke, and able to understand and speak Spanish. The professionals’ group included medical doctors (with at least 6 years of training), family doctors (with an additional 4‐year specialist training), nurses (with at least 5 years of training), primary care technicians (with at least 2 years of training), officials of the district directorates or zonal coordinating offices involved, and national directors of the Quality Directorate of the Ministry of Public Health. All of them had at least 5 years of experience in the public sector and worked in rural areas of Zone 3.

### 2.3 Focus groups

Three focus groups were conducted with 10 patients and 18 professionals (physicians, nurses, and primary care technicians in direct contact with indigenous populations) to capture information from different perspectives and experiences and to draw on the care process's milestones. One focus group was performed with patients in August 2019, another with professionals in the care of indigenous patients in January 2021, and the last with managers and middle management of the Quality Directorate of the Ministry of Public Health in February 2021. Using this information, ‘questionnaire 0’ for the Delphi technique was prepared. The time lag between sessions was due to the COVID‐19 pandemic.

Face‐to‐face interviews were conducted with the patients. A purposive sampling strategy^14^ was used to recruit individuals with type 2 diabetes who were interested in discussing their experiences. Participants were contacted through phone calls made by professionals at the health centres they usually attended. On arrival, the aim of the focus groups was explained, and the patients decided if they wished to participate. The focus group was performed in a private room in the same health centre attended by participants to provide a familiar environment to generate trust. Informed consent was obtained before the focus group, and permission for audio recording was also obtained. The participants’ anonymity and data confidentiality were respected. They were also informed that they had the right to leave the focus group at any time without giving a reason. None of the participants declined the invitation to participate in the focus groups or interviews.

The focus group with professionals was conducted through telematics to comply with national regulations and biosecurity measures during the COVID‐19 pandemic. The research group previously developed and agreed to the session script (Supporting Information: File 1). The sessions lasted 90 min and were initiated after obtaining the consent of all participants. Each focus group was recorded and transcribed for later analysis according to the emerging themes of the previously established scripts. During an online session, participants were asked to generate ideas through the Mural application (Tactivos).

### 2.4 Qualitative analysis

The focus group was audiotaped and transcribed verbatim. We applied Braun and Clarke's^15^ thematic analysis, which comprises the following steps: (1) familiarizing oneself with the data, (2) generating initial codes, (3) searching for themes, (4) reviewing themes, (5) defining and naming themes, and (6) producing the report. After familiarizing themselves with the information obtained from all groups, two authors independently elaborated the categories of analysis to codify the ideas contributed by each group in an orderly manner. In the case of inconsistencies in coding, a third researcher intervened to facilitate consensus. The codes were then reviewed and revised by a third author. The authors then analysed the consistency (repetitions) of the ideas contributed by patients, professionals, and managers in each category. Discussions were held to ensure the appropriate triangulation of ideas between patients, professionals, and managers, which led to identifying quality criteria and essential attributes. Finally, these criteria were ordered according to the classification proposed by Donabedian^16^ of structure, process, and results, giving rise to ‘questionnaire 0’ used in the Delphi technique.

### 2.5 Delphi study

The Delphi technique seeks the opinions of a group of experts to assess the extent of agreement and resolve disagreements.^17^ This technique was used to identify the quality criteria that, in the opinion of clinicians and managers, would make it possible to monitor the results of healthcare interventions in the study population. The relevance, importance, and feasibility criteria were evaluated on a Likert scale ranging from 0 (*not at all important or not feasible*) to 10 (*very important or feasible*). The Delphi technique was performed using an online platform designed for this study. This web‐based software allowed the generation of personal links for each participant. Only healthcare professionals were included in the Delphi study.

The Delphi process comprised two voting rounds, conducted 45 days apart. Following the focus groups and qualitative analysis, Questionnaire 0 was developed. The first round included ‘Questionnaire 0’, which comprised 39 indicators into six categories. The participants had to vote using the previously described Likert scale for each indicator. A free‐text response was available to the participants in the survey, providing the opportunity to propose or modify indicators. Indicators that scored ≥6 were included in the final proposal (high consensus for inclusion). Indicators with scores ≤4 were discarded (high consensus for not considering). Indicators with scores of 5 or 6 were assessed in the second round. The research group and frontline professionals determined their relevance, priority, and feasibility if a consensus was not reached.

In Round 2, each participant received an individualized survey comprising 17 indicators. It included 13 indicators from Round 1 which did not reach consensus and were presented alongside the participants’ responses from Round 1. Round 2 also included four new indicators derived from free‐text responses from Round 1. Furthermore, the free‐text answers from Round 1 provided clarity on a statement. This was subsequently added as a new statement in Round 2. There was no option for free‐text responses in Round 2. Indicators that scored ≥5 were included in the final proposal (high consensus for inclusion).

### 2.6 Consensus conference

Following the Delphi study, the research team, two experts in quality and patient safety, middle management, health professionals, and managers of a health district providing direct care to the indigenous population analysed the study results. It generated a proposal for indicators after considering their relevance, usefulness, and feasibility.

## 3 RESULTS

**Figure 1:**
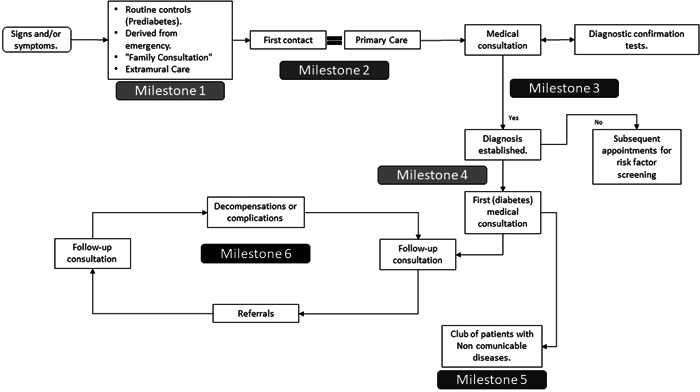
Milestones in the care process for people with diabetes. Milestone 1: Initial consultation; Milestone 2: First contact in primary care; Milestone 3: Complementary diagnostic tests; Milestone 4: Entry into the national health system for the control of NCDs; Milestone 5: Permanence in the NCD Club; Milestone 6: Permanence in the national health system for the management of NCDs. NCD, noncommunicable disease.

The qualitative analysis of the focus group discussions identified six milestones in diabetes care (Figure 1), and factors that conditioned the care were classified into six dimensions (Table 1). This analysis allowed the identification of criteria for adequate care for each milestone along the pathway.

**Table 1:** Categories of analysis for each of the three groups of participants

Categories	Patients	Professionals	Managers
Cosmovision		Cosmovision sometimes can delay diagnosis and diminish confidence in medicine.	Lack of confidence in the health system.
		Lack of access to information about the disease and healthy diet.	Provide more health education by trained health centre staff (Primary Care Technicians).
		Difficult access to healthcare resources.	
	‘According to me, I am fine, but according to the doctors, I am not fine’, but it does not hurts me.	Lack of acceptance of the disease (awareness of being sick).	
	It was challenging to have to make two different meals in our house.	Socioeconomic difficulties of patients that compromise healthy eating.	Diabetic eating style.
		Late diagnosis in hospitals (with the appearance of complications).	Access in hospitals. The necessary information is not available.
	Sometimes it is difficult to get to the health centre.	Difficulties in transporting patients.	Geographic areas of difficult access.
Accessibility and adaptability to the cosmovision		Lack of health promotion and disease prevention measures.	Lack of protocols adapted to intercultural practices.
	Laboratory tests appointments are available only one day a week, and sometimes they are not.	Lack of flexibility in the management of laboratory and medical appointments.	Limited access to consultation and laboratory services.
		Difficulties in communication (doctor‐patient relationship).	Health centres must have at least one member who speaks the native language.
	When I ran out of money, I stopped going to the doctor.	Social and economic vulnerability of the communities.	Economic Vulnerability.
Equipment and resources		Lack of specialists (multidisciplinary team)	Lack of resolution capacity of various services in health facilities.
		Delays in patient follow‐up after the onset of complications (psychology and physical therapy).	Support from psychology, especially for the accompaniment of patients in a situation of abandonment, elderly and/or vulnerable adults.
		Failures in specialist support to general practitioners in remote areas	Strengthen the follow‐up of consultations by specialists complementary to the process.
	Sometimes we never get the lab results.	Absence or lack of medication.	Increased access to diagnostic tests.
		Scarce laboratory tests.	
	‘When I arrived at the health centre, they told me, ‘diabetics aside’, that affected me the most’.	Failures in communication between hospitals and health centres and the different health services.	Ethnic discrimination.
			Adaptation of spaces according to the needs of the users.
Community care (EAIS/Local Health Committee)		Lack of updating of health professionals (doctors, nurses, Primary Care Technicians).	Reinforce the attention of family visits.
			Involve the health committee in talks about healthy eating habits.
			Strengthen the participation of the local health committee.
			Improve the planning of extramural work.
	Meetings, such as support groups, are good for us.		Establish a connection between health promoters and community leaders.
Quality culture	Sometimes the doctor does not have enough time, but most of us wait since we know each other.	Short consultation time per patient.	Systematic use of telemedicine programs through satellite calls has not been considered.
	They do not tell you clearly what you can and cannot eat.	Lack of health education (compromising self‐care and adherence to treatment).	Complex therapeutic compliance on the part of the indigenous population (due to various factors).
Results		Include diagnostic criteria for prediabetes to carry out prevention processes.	To create a scorecard with established quality standards.
			Measure health education outcomes.

The professionals’ emphasized that the cosmovision conditioned the relationship and the first approach between the indigenous population and the health sector (Milestone 1). Patients’ beliefs and lack of health education could also delay their first contact.

We went to Salcedo to have a ‘cleanse’ with a guinea pig. And there, he told me, why didn't you come quickly? This is diabetes. He told us that we had to go quickly to a doctor. (Female patient, 64 years)

I had some symptoms, mainly thirst, and since I had no knowledge, I thought it was from playing volleyball, and when I got home in the afternoon, I kept drinking soda. (Male patient, 53 years)

They usually have a diet rich in carbohydrates, and because of its cheaper cost, they tend to drink a lot of soda. (Primary care technician)

Managers mentioned that it could be due to a lack of confidence in the indigenous population in the health system. Both managers and professionals commented on the difficulties patients sometimes face when accessing health due to geographical challenges and the occasional lack of public transport. In most cases, patients’ first interaction with the national health system occurs through the first level of care. However, professionals mentioned that due to the delay, the diagnosis was made in hospitals after the appearance of complications. In this first encounter, patients were highlighted as key factors in treatment, flexibility, and responsiveness (Milestone 2).

On some occasions, patients complain about not being able to show up on time for a scheduled lab or medical appointment because they miss the bus and the next one takes an hour. (Medical doctor)

In the emergency room, they told me that I had diabetes, and they admitted me for 10 days and put me on insulin for a month. (Female patient, 51 years)

Delays in care, availability of complementary tests, waiting times, and access to these tests were crucial factors (Milestone 3).

The only thing I have complained about is the laboratory tests. As we send them from here, the sample and sometimes the results do not arrive. (Female patient, 49 years)

Once the diagnosis was made, entry into the national health system to control chronic diseases was highlighted as a key moment because assertiveness would condition their future permanence (Milestone 4).

When I arrived at the health centre, they told me, ‘diabetics aside,’ that affected me the most. (Male patient, 47 years)

I almost had a heart attack when they gave me the news because they directly tell you that you have diabetes and there is no cure. (Female patient, 55 years)

In Ecuador, one of the strategies used at the first level is the ‘Club of patients with chronic diseases’. This group of patients is usually led by a doctor or nurse responsible for providing health education to the patients and sometimes for recreational and awareness‐raising activities. Patients emphasized that adequate treatment, information, support, and promotion of healthy habits, well‐being, and quality of life were fundamental for their permanence in the club (Milestone 5).

I always drank soda, and even then, I was still thirsty. (…) I am getting better, and I feel good little by little. Now it is forbidden to drink soda. (Female patient, 61 years)

I have now lowered my glucose completely. I keep checking myself, and sometimes it goes down to 110 or 90, but the doctor told me that I could not lower it that much because I felt like I was fainting, and I could not get up. Now I am at 140. (Male patient, 47 years old)

(When asked about the Club) Yes, of course, because it's like a support group. (Male patient, 51 years)

Finally, participants mentioned continuity of care and availability of resources and supplies as critical factors for patients’ permanence in the national health system (Milestone 6).

**Figure 2:**
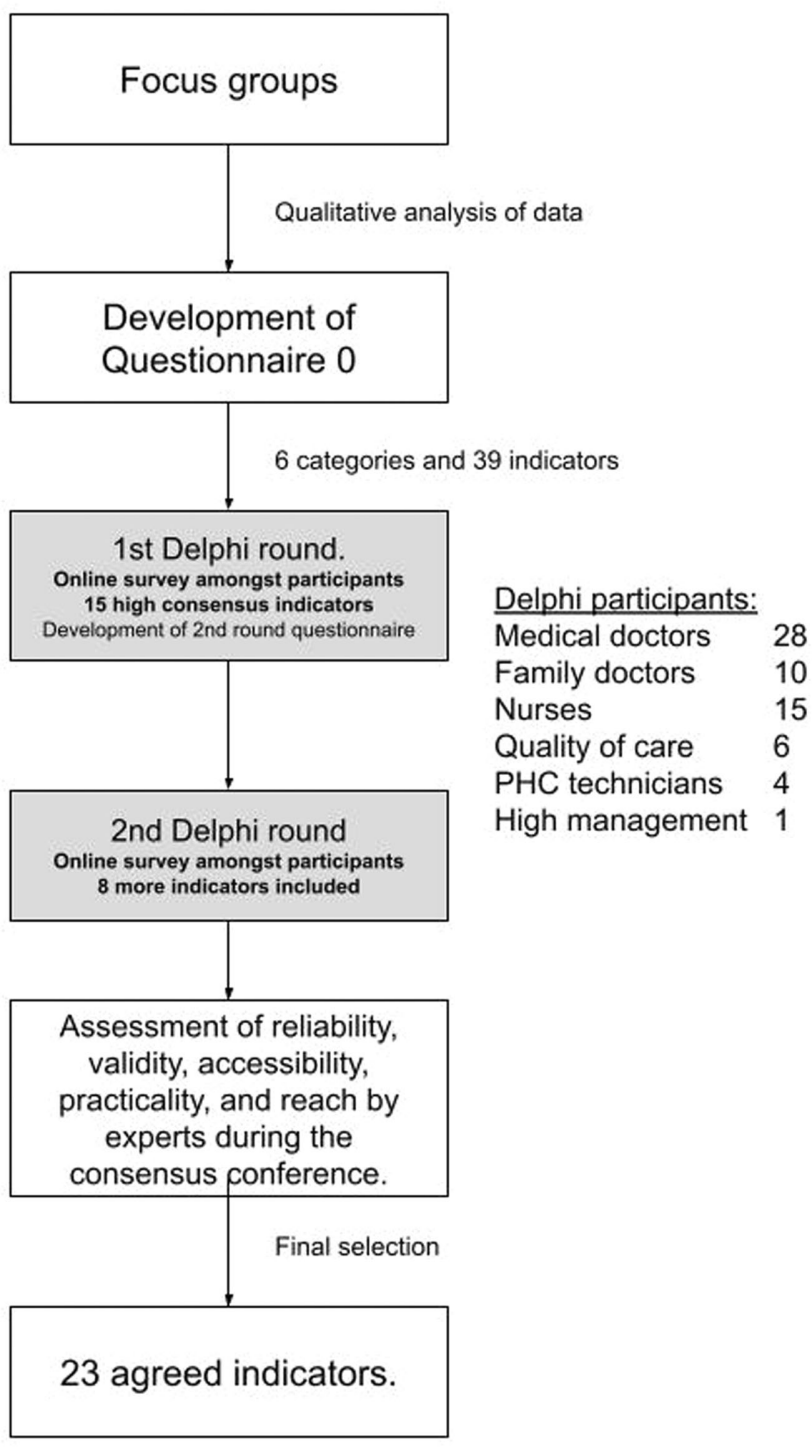
Flowchart of the study

Questionnaire 0, used in the Delphi technique, included a criteria total of 39 quality indicators grouped into six dimensions. A total of 64 professionals responded in the first round (90% response rate) and 34 in the second round (53% response rate) (Table 2). The Delphi comprises medical doctors, family doctors, nurses, primary care technicians, managers, and quality of service provision professionals. Of these professionals, nine were indigenous. In the first round, 15 criteria were agreed upon (high level of consensus), and 11 were discarded (low level of consensus). Thus, in the second round, 13 criteria from the first wave were included (doubtful consensus), and participants added four new criteria. Eight indicators were included at the end of the second round (Figure 2).

**Table 2:** Participants in the two rounds of the Delphi technique

	1st round (*n* = 64)	2nd round (*n* = 34)
Sex		
Female	42	23
Male	22	11
Ethnicity		
Indigenous	9	9
Other ethnicities	55	25
Years of professional experience		
Less than 1 year	14	1
Between 1 and 2 years	9	1
Between 2 and 5 years	12	6
More than 5 years	6	12
More than 10 years	23	14
Professional category		
Medical doctors	28	26
Family doctors	10	6
Nurse	15	1
Primary Care Technicians	4	0
Managers	1	0
Quality or service provision professionals	6	1
Population		
Rural	49	28
Urban	15	6

Following the results of the Delphi technique, a consensus conference was held by the CORE group and a working group of professionals and middle managers caring for the indigenous population. This conference was held in a health district in the central highlands of Ecuador. During this session, each indicator's reliability, validity, accessibility, practicality, and reach and the votes received during the Delphi technique were evaluated. This group also agreed to include these five indicators with the doubtful consensus of the second round. Finally, the structure (7), process (5), and result (11) indicators were developed and distributed in the six dimensions previously identified. The indicators formed a dashboard to monitor the care received by indigenous people with diabetes (Tables 3, 4, 5).

**Table 3:** Structure indicators

Dimension	Indicator	Mean	VC
Quality culture	The centre has a strategy to avoid ethnic discrimination.	7.54	0.4
Quality culture	The centre has a strategy to avoid gender discrimination.	7.37	0.4
Quality culture	There is a protocol for coordinated care between the health centre and its referral hospital.	6.67	0.4
Quality culture	There is a prevention strategy (that includes prediabetes).	6.63	0.4
Cosmovision (literacy and social, economic, and cultural aspects)	The ratio of diabetic patients enroled. The number of patients enroled divided by the theoretical incidence projection.	6.48	0.43
Accessibility and adaptability to the cosmovision	Public transportation is adjusted to the scheduled consultation and laboratory schedules for people who must travel from other cities and towns	5.48	0.49
Resources and equipment	There is a support program for primary care professionals by specialists through telemedicine or satellite telephones.	4.98	0.70

Abbreviation: VC, coefficient of variation.

**Table 4:** Process indicators

Dimension	Indicator	Mean	VC
Cosmovision (literacy and social, economic, and cultural aspects)	The proportion of scheduled appointments missed by patients (living in communities or areas difficult to access).	6.67	0.5
Community care (EAIS/Local Health Committee)	The proportion of home visits made by EAIS to patients in hard‐to‐reach areas.	6.23	0.4
Dashboard‐ results	The proportion of patients with an individualized therapeutic plan.	6.19	0.5
Accessibility and adaptability to the worldview	Average waiting time to make a medical appointment at the health centre (waiting times).	5.92	0.5
Community care (EAIS/Local Health Committee)	The number of persistent patients with an orientation by a community leader as a Health Promoter (‘Expert Patient’ Program).	5.53	0.6

Abbreviation: VC, coefficient of variation.

**Table 5:** Results indicators

Dimension	Indicator	Mean	VC
Cosmovision (literacy and social, economic, and cultural aspects)	The proportion of patients with diabetes at risk by economic vulnerability.	7.03	0.39
Cosmovision (literacy and social, economic, and cultural aspects)	The proportion of patients who have received health education.	6.80	0.30
Accessibility and adaptability to the cosmovision	The proportion of patients with adequate control (subsequent appointments). Persistence.	6.32	0.38
Cosmovision (literacy and social, economic, and cultural aspects)	The proportion of patients withdrawing complete medication at the health centre pharmacy.	6.31	0.37
Dashboard—Results	The proportion of patients with a positive experience of the care received (EPD Questionnaire).	6.30	0.42
Dashboard—Results	The proportion of patients in the therapeutic range (glycosylated haemoglobin result) in the last 90 days.	6.29	0.48
Dashboard—Results	The proportion of laboratory results reported in less than 72 h.	6.27	0.46
Cosmovision (Literacy and social, economic, and cultural aspects)	The proportion of family members or companions who have received health education.	6.17	0.37
Dashboard—Results	The proportion of patients presenting to the emergency department due to decompensation.	5.93	0.61
Dashboard—Results	Prevalence of chronic complications of diabetes.	5.76	0.57
Accessibility and adaptability to the cosmovision	According to nutritional survey results, the proportion of patients with a nondiabetic diet (number of times per week eating vegetables, chicha, and other high carbohydrate foods).	5.74	0.46

Abbreviation: VC, coefficient of variation.

## 4 DISCUSSION

To the best of our knowledge, this is the first study to include indigenous populations’ perspectives on diabetes control in Ecuador. The agreed set of indicators consisted of the usual indicators in evaluating outcomes that can be found in clinical guidelines (e.g., the proportion of patients in the therapeutic range in the last 90 days). Nevertheless, other indicators focused on how health education is approached, patient activation in self‐care, and the organization of the care process, emphasizing the cosmovision of the disease and the reality of the socioeconomic situation of the indigenous population that conditions their access to a healthy diet or health.

As pointed out by previous authors, the focus should be on building a therapeutic relationship with indigenous people with diabetes rather than a singular emphasis on achieving management targets.^18^ As commented by the participants of this study, there was a lack of confidence in indigenous populations in the healthcare system, which seems to systematically affect other contexts.^19^ The current poor success in achieving management targets highlights the limitations of health services that are not relevant to indigenous populations’ social and cultural context.

This study also confirmed that the perspectives of patients and professionals did not coincide entirely. Furthermore, it highlighted the need for healthcare workers to make additional efforts to adjust to the needs and expectations of patients and their families. This aspect is crucial to achieving the objectives of Ecuador's care process, which includes person‐centred care in the terms proposed by the WHO.^20^, ^21^

This set of indicators identified in our study helps shift the focus of outcome assessment in diabetes treatment from the pathology to the person, and in this case, to a group of people who share values, beliefs, and customs that require differentiated attention to achieve the intended outcomes.^22^

There is a considerable inequity in the health of the indigenous populations globally, who are the world's most marginalized population groups.^23^, ^24^ There is a difference of up to 12 years in life expectancy between indigenous and nonindigenous people.^25^ A review compared the management of patients with diabetes (indigenous and nonindigenous) in five high‐income countries and identified potential areas for improvement and existing gaps.^26^ Ecuadorian health authorities can use this set of indicators to complement process measures currently planned to control metabolic and chronic diseases. Locally, health authorities should engage with community leaders to establish effective prevention strategies that are essential and grounded in the community's specific social, cultural, and health service contexts. Prediabetes is a significant opportunity to prevent or delay diabetes through healthy behavioural interventions.

However, the prevalence of diabetes is similar in other Latin American countries.^27^ It can be assumed that some of the characteristics identified in this population might also be similar. A study across Brazil, Peru, Colombia, Bolivia, Argentina, Chile, Paraguay, and Ecuador showed that the main barriers to accessing primary healthcare services were the difficulty in reaching the healthcare facilities, difficulty in communicating with healthcare professionals, inadequate transportation to the healthcare units, lack of epidemiological data on indigenous villages, lack of information regarding local indigenous cultures, and fear of discrimination or humiliation on the part of indigenous patients.^28^ All these limitations were addressed in this study. This proposal of indicators may also be applicable in other countries of the Andean Community and Latin America, which fit better than the indicators reported for high‐income countries with different socioeconomic conditions. Finally, projects to improve the quality of life of this population financed by entities such as the Inter‐American Bank or the World Bank or the Pan American Health Organization activities in this region may benefit from having structure, process, and outcome indicators adapted to this reality instead of measures imported from other contexts.

The greatest usefulness of these indicators is when they are used within the framework of a cycle of improvement, providing information on the effectiveness of the designed interventions. This framework currently implemented in Ecuador would benefit when adjusted to the local context and its different population groups. In this sense, it is essential to emphasize that the selected indicators meet the premise that they can be easily obtained from current information systems and therefore add to their relevance and feasibility. As mentioned earlier, these indicators could also benefit the Andean community in other countries, but their adaptability should be assessed before application in different contexts.

This study had some limitations. Using electronic means of communication instead of face‐to‐face sessions led to a loss of detail and valuable information in qualitative research. However, a key strength of this study is that we sought to include patients and healthcare workers in direct contact with the study population to improve and personalize the results. Another limitation is the time lag between patients’ focus groups and healthcare workers’ videoconferences, owing to COVID‐19 restrictions. A smaller number of nurses participated in the second round of the Delphi study than in the first due to connectivity difficulties in certain areas. Due to technological barriers and limited Internet access, patients or their families could not be included during the Delphi study. Nevertheless, the final meeting allowed for an attempt to seek clarification. There is inherent variability within the territory in access to laboratory tests and in the organization of public transport that facilitates access to health centres. These aspects should be considered when extrapolating results to other contexts.

## 5 CONCLUSION

There was a consensus on the set of indicators among all the participants, patients, direct healthcare professionals, and managers, strengthening the results. These indicators have considered the feasibility and relevance and aimed to achieve comprehensive person‐centred care for diabetes among the indigenous population in Ecuador and possibly the region.

## AUTHOR CONTRIBUTIONS

**Jimmy Martin‐Delgado**: Conceptualization; methodology; project administration; funding acquisition; roles/writing – original draft; visualization. **Carla Tovar**: Investigation; data curation; validation. **Israel Pazmiño**: Investigation; validation**. Amelia Briones**: Investigation; validation**. Irene Carrillo**: Conceptualization; funding acquisition. **Mercedes Guilabert**: Conceptualization; funding acquisition**. José Joaquín Mira**: Conceptualization; methodology; project administration; funding acquisition; roles/writing – original draft; supervision**. All authors**: Writing – review & editing of the final draft.

## CONFLICT OF INTEREST

The authors declare no conflict of interest.

## ETHICS STATEMENT

The study was approved by the Ethics Committee of the Hospital Clínica Kennedy of Guayaquil, Ecuador (HCK‐CEISH‐19‐0041). Informed consent was obtained from all subjects involved in the study. I confirm all patient/personal identifiers have been removed or disguised so the patient/person(s) described are not identifiable and cannot be identified through the details of the story.

## Supporting information

Supplementary information.

## Data Availability

The data that support the findings of this study are available on request from the corresponding author. The data are not publicly available due to privacy or ethical restrictions.
