# Supplementary material for: Indicators for adequate diabetes care for the indigenous communities of Ecuador
Source: Health Expect. 2022 Oct 31;25(6):3315–25. doi: 10.1111/hex.13643 (PMC9700129; doi:10.1111/hex.13643)
Supplement: Supplementary file 1 — Supplementary information. [file HEX-25--s001.docx]

Script

Brief presentation.

Group and research objectives.

Theoretically, we have represented the care process in 6 milestones (key moments). Ask whether to modify the sequence. Ask following the child of the process if something is not OK. At each Milestone ask for main barriers and constraints. Each person writes down what he/she considers most relevant and uploads it on the Mural Application.

1. Gateway, access to the system (primary, specialist, emergency)
2. Characteristics of the initial consultation, how it is initiated, which professional profile usually identifies a possible case, what he/she usually does and what he/she should do.
3. How often do you usually see patients after diagnosis and treatment? (and level of care)
   1. What is the professional patient ratio or how many consultations per day?
   2. How much consultation time is usually available?
4. At what point in the care process is the most critical, where it is important to have the appropriate means available.
   1. Availability of complementary tests and medicines.
5. Participation in diabetic education programs (prescription, recommendation)
6. Existence of contradictory information between the different levels of care provided by professionals.
7. Perception of variability in clinical practice (use of standardized protocols in the management, diagnosis and treatment of diabetic patients).
8. Within the care provided to diabetic patients, what are the most valued aspects, from the patient's point of view?
9. What aspects should patients look for in order to assess that the health care has been correct?
10. Who is the referral for this type of patient (endocrinologist, internal medicine, primary care, nursing? )?
11. Most frequent complaints/complaints (if any) that you usually deal with
